# Transcriptomic Analysis of *Takifugu obscurus* Gills under Acute Hypoxic Stress

**DOI:** 10.3390/ani13101572

**Published:** 2023-05-09

**Authors:** Huakun Zhang, Run Li, Yaohui Wang, Jinxu Zhou, Hao Xu, Meng Gou, Jianhua Ye, Xuemei Qiu, Xiuli Wang

**Affiliations:** 1 College of Fisheries and Life Science, Dalian Ocean University, Dalian 116023, China; 2 Key Laboratory of Pufferfish Breeding and Culture in Liaoning Province, Dalian Ocean University, Dalian 116023, China; 3 Jiangsu Zhongyang Group Company Limited, Nantong 226600, China; 4 College of Life Science, Liaoning Normal University, Dalian 116081, China

**Keywords:** *Takifugu obscurus*, acute hypoxic stress, transcriptomics, gill

## Abstract

**Simple Summary:**

*Takifugu obscurus* is an economically important fish because of its fast growth rate, tender meat, and high nutritional value. The rapid development of the aquaculture industry in recent years has led to the increasing persistence of hypoxia in aquaculture water systems. Dissolved oxygen is an important factor for fish survival, and low oxygen stress can cause slow growth and reduced immunity of fish. Gills are the main respiratory organs of fish and are greatly affected by changes in dissolved oxygen levels. In this study, the transcriptomic responses of *T. obscurus* gill tissues were compared under acute hypoxic stress relative to normoxic and reoxygenated conditions to identify differentially expressed genes associated with hypoxia and understand the adaptive changes of these genes in fish responses to hypoxia. This study provides new insights into the molecular mechanisms underlying the responses of *T. obscurus* to hypoxic stress.

**Abstract:**

*Takifugu obscurus* has relatively small gills and gill pores, leading to a relatively low respiratory capacity and increased vulnerability to low dissolved oxygen (DO) levels compared to other fish. To investigate the responses of *T. obscurus* to acute hypoxic stress, high-throughput-sequencing-based transcriptomic analyses were conducted here to assess the responses of *T. obscurus* gills to acute hypoxic stress. Three environmental conditions were compared including normoxia (DO: 7.0 ± 0.2 mg/L), hypoxic stress (DO: 0.9 ± 0.2 mg/L), and reoxygenation (4, 8, 12, and 24 h after return to normoxia) conditions to identify differentially expressed genes (DEGs) responsive to hypoxia. A total of 992, 877, 1561, 1412, and 679 DEGs were identified in the normoxia and reoxygenation for 4, 8, 12, and 24 h groups in comparison to the hypoxia groups, respectively. The DEGs were primarily associated with oxidative stress, growth and development, and immune responses. Further functional annotation enrichment analysis of the DEGs revealed that they were primarily related to cytokine–cytokine interactions, transforming growth factor β receptor (TGF-β), cell adhesion molecules (CAMs), the vascular endothelial growth factor (VEGF) signaling pathway, and the mitogen-activated protein kinase (MAPK) signaling pathway. These results provide new insights into the physiological and biochemical mechanisms of *T. obscurus* adaptations to hypoxic stress. Furthermore, these results provide a framework for future studies into the molecular mechanisms of hypoxia tolerance and the healthy culture of *T. obscurus* and other fish.

## 1. Introduction

Dissolved oxygen (DO) is an essential nutrient in environments that affects the growth, metabolism, and other physiological activities of aquatic animals, thereby acting as a major limiting factor in aquaculture and being a basic necessity that aquatic animals require for survival [1]. The DO concentrations in water generally depend on many factors, including the photosynthetic activities of phytoplankton, respiration by aquatic organisms, and the diffusion of oxygen into water from the atmosphere. Global and seasonal water hypoxia have occurred in recent years due to increased water eutrophication. These low-oxygen environments have deleterious effects on fish behavior, growth, physiology, and disease resistance, even leading to fish mortality [2]. Generally, fish require DO concentrations above 5 mg/L in the water column, while feeding is significantly reduced below 2 mg/L, and fish appear to float their heads or even die of suffocation at levels below 1 mg/L [3]. During typical high-density and shallow pond water cultivation, high culture densities, baiting, and the relative closure of water bodies are more likely to lead to fish hypoxia, thereby representing a major factor that limits the cultivation of *T. obscurus*. Consequently, additional research is needed to understand hypoxic in stress of *T. obscurus* to ensure the sustainable development of the pufferfish aquaculture industry.

The pufferfish *T. obscurus* (superclass *Osteichthyes*, order *Plecoptera*, family *Tetraodontiformes*) is a marine and freshwater migratory fish that is primarily distributed in offshore Chinese oceans and in the middle and lower reaches of the Yangtze River. Because of its delicious meat, low fat content, and high protein content, it is known as one of the three delicacies of the Yangtze River, and it is native to China. The fish are often active in the lower and middle waters, and they exhibit a weak respiratory capacity, leading to higher susceptibility to low DO concentrations compared to other fishes [1]. The fish also exhibit aggressive and voracious habits, high protein consumption, and physiological activities that require high oxygen consumption, thereby leading to a high DO asphyxiation point [4].

Many studies have evaluated the mechanisms of hypoxia tolerance in fish using transcriptomic techniques, including in *Megalobrama amblycephala* [5], *Aristichthys nobilis* [6], and *Salmo salar* [7], but few have evaluated the adaptations of *T. obscurus* to hypoxia. Gills are the main organ involved in gas exchange and osmoregulation in fish and are characterized by large surface areas and sensitivities to changes in water conditions, thereby representing a key factor related to fish adaptation to external environments [8]. In this study, the effects of acute hypoxic stress on the gill tissues of *T. obscurus* were investigated with transcriptomic techniques, the identification of differentially expressed genes (DEGs), and the identification of biological pathways associated with hypoxia tolerance. The specific objective of this study was to better understand the adaptive mechanisms of fish to hypoxic stress.

## 2. Materials and Methods

### 2.1. Ethics Statement

In the study, all fish were handled with strict accordance to the Chinese legislation on scientific procedures on living animals. The study protocol was approved by the ethics committee at Dalian Ocean University (DLOU). Before handling fish, they were anesthetized with 100 ng/mL of tricaine methanesulfonate (MS-222, Sigma, St. Louis, MO, USA), and all efforts were made to minimize suffering. Field studies did not involve endangered or protected species. The Ethics Approval Code is DLOU-2021-025.

### 2.2. Experimental Fish

This study randomly selected 300 healthy *T. obscurus* (with an average weight of 91.6 ± 22.9 g) from the *T. obscurus* breeding farm of Jiangsu Zhongyang Co. in Hai’an, Nantong, Jiangsu, China, for investigation. The fish were then transferred to experimental pools with a water temperature of 24 ± 1 °C and acclimated for one week. During this period, DO levels in the experimental pools were maintained at 7.0 ± 0.2 mg/L by adjusting aeration, and the fish were fed with compound feed and small fry. Prior to experimentation, the fish were fasted for 24 h and were not fed throughout the experiment.

### 2.3. Experimental Design and Sample Collection

From the experimental pool of 300 fish, 120 fish were selected for a pre-experiment to identify the hypoxic threshold, at which fish reach their limit of hypoxia tolerance and lose equilibrium. The hypoxic threshold was determined to be 0.9 ± 0.2 mg/L. In the subsequent experiment, 40 fish were randomly selected and transferred to a 100 L tank with a water temperature of 24 ± 1 °C. The experimental tank was tightly sealed with film, and real-time decreases in DO content were monitored. Six fish were then randomly selected at each of the following time points: normoxia (DO: 7.0 ± 0.2 mg/L); acute hypoxic stress (DO: 0.9 ± 0.2 mg/L); and 4, 8, 12, and 24 h after the water returned to normoxic levels (DO: 7.0 ± 0.2 mg/L). After quickly anesthetizing the sampled fish with MS-222 (300 mg/L), the gills were carefully removed. Gill samples from different fish at each timepoint were pooled and transferred to centrifuge tubes (each sample containing tissues from six fish), and five biological replicates were used for each time point. The gill tissue samples collected under normoxic conditions were labeled as GC (normoxia control group); hypoxic samples were labeled as GH (hypoxia experimental group); and the reoxygenation groups at 4 h, 8 h, 12 h, and 24 h were labeled as GR_4, GR_8, GR_12, and GR_24, respectively. Subsequently, these gill samples were immediately frozen in liquid nitrogen and stored at −80 °C. The experiments were repeated three times for accuracy.

### 2.4. RNA Extraction, Library Construction, and Transcriptomic Sequencing

RNA sample extraction, library construction, and library sequencing were conducted by Beijing Novogene Technology Co. (Beijing, China). The purity, concentration, and integrity of RNA samples were evaluated with an RNA Nano 6000 Assay Kit and a Bioanalyzer 2100 system (Agilent Technologies, Santa Clara, CA, USA) to ensure that only high-quality samples were used for transcriptome sequencing. Total extracted RNAs were used as input for RNA sample preparations. To summarize, firstly, enriched mRNAs with polyA tails were enriched using Oligo (dT) magnetic beads. Next, the resulting mRNAs were randomly interrupted with divalent cations in fragmentation buffer. The fragmented mRNAs were utilized as a template, and random oligonucleotides acted as primers to synthesize first-strand cDNAs with the M-MuLV reverse-transcriptase system (Agilent Technologies, Santa Clara, CA, USA). Then, RNA strands were degraded using RNaseH, and second-strand cDNAs were synthesized with dNTPs and the DNA polymerase I system. The purified double-stranded cDNAs were end-repaired, A-tail ligated, and ligated with sequencing junctions. cDNAs with lengths of about 370–420 bp were screened with AMPure XP beads and amplified using polymerase chain reaction (PCR). The PCR products were again purified using AMPureXP beads to obtain final libraries. After libraries were constructed, they were initially quantified using a Qubit 2.0 Fluorometer and then diluted to concentrations of 1.5 ng/μL. The insert sizes of the libraries were then evaluated using an Agilent 2100 bioanalyzer. Following confirmation of appropriate insert sizes, the effective concentrations of the libraries were quantified with qRT-PCR (quantitative reverse transcription PCR) to ensure that the effective concentrations of the libraries were >2 nM.

After library validation, the clustering of index-coded samples was performed on a cBot Cluster Generation System using the TruSeq PE Cluster Kit v3-cBot-HS (Illumina, San Diego, CA, USA) according to the manufacturer’s instructions. After cluster generation, library preparations were sequenced on an Illumina Novaseq platform, and 150 bp paired-end reads were generated.

### 2.5. Data Filtering, Read Mapping, and Differential Expression Analysis

Raw data (raw reads) in the fastq format were transformed using CASAVA base identification from image data measured using the high-throughput sequencer. To ensure the high quality and reliability of data analysis, reads containing adapters, reads with undetermined bases, and low-quality reads (i.e., those with Qphred ≤ −20 bases accounting for >50% of the entire read length) were removed from the raw data to obtain clean sequence data. In addition, Q20, Q30, and GC levels were calculated for the clean sequence dataset. All subsequent analyses were conducted using the high-quality clean sequence data.

The reference genome and gene model annotation files were obtained from the genome website. HISAT2 v2.0.5 was used to build an index of the reference genome, and then the paired-end clean reads were compared with the reference genome to obtain the positioning information of the clean reads on the reference genome.

Differential expression analysis between the two treatment combinations was performed using the DESeq2 software package (version 1.20.0). DESeq2 statistically analyzes the differential expression of numerical gene expression data using a model based on negative binomial distributions. The Benjamini–Hochberg method was also used to adjust the resulting *p*-values to control for error rates. DEGs were defined on the basis of log2 (FoldChange) ≥ 1 and *p*-values ≤ 0.05. Gene Ontology (GO) enrichment analysis of DEGs was then conducted using the clusterProfiler (3.8.1) software program, while correcting for gene length bias. GO terms with padj < 0.05 were considered significantly enriched. In addition, the enrichment of DEGs based on comparison to the Kyoto Encyclopedia of Genes and Genomes (KEGG) pathway database was evaluated using the clusterProfiler (3.8.1) software program.

## 3. Results

### 3.1. Summary of Sequencing Data Quality

Transcriptomes of *T. obscurus* gill tissues were analyzed from normoxic (GC), hypoxic (GH), and reoxygenation (GR_4, GR_8, GR_12, GR_24) groups. After filtering the raw data, checking the sequencing error rate, and checking the GC content distribution, the clean reads used for the subsequent analysis were obtained. The data are summarized in Table 1. The percentage of Q30 bases were all above 92.97% for each library. Thus, the sequencing data quality was good and could be used for subsequent analyses.

### 3.2. Read Mapping

The HISAT2 v2.0.5 software was used to obtain the localization information of reads on the reference genome, and then the clean reads were accurately compared with the reference genome. The samples were compared with the reference genome, as shown in Table 2.

### 3.3. DEG Analysis

The number of DEGs in the GH group and each of the other groups is shown in Table 3. A total of 992 DEGs were identified for the GC and GH comparison group, and a total of 877 DEGs were identified in the GR_4 and GH comparison group. In addition, 1561 DEGs were identified in the GR_8 and GH comparison group. In the GR_12 and GH comparison group, 1412 DEGs were identified. Lastly, 679 DEGs were identified in the GR_24 and GH comparison group.

**Figure 1:**
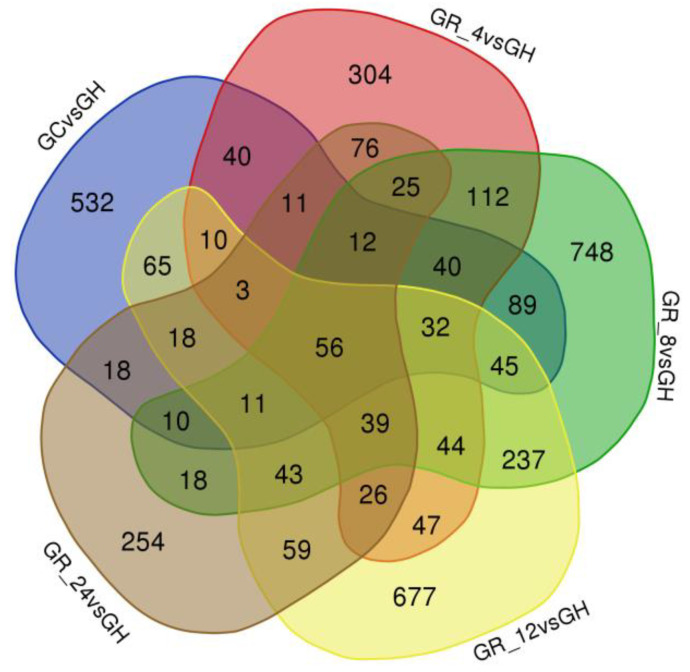
Venn diagram of differentially expressed gene datasets among gill tissues of *Takifugu obscurus* that were recovered under different oxygen availability conditions. GC is the normoxia control group; GH is the hypoxia group; and GR_4, GR_8, GR_12, and GR_24 are the reoxygenation groups at 4 h, 8 h, 12 h, and 24 h, respectively.

In addition, the gene co-expression between different DEG datasets was investigated to identify shared and unique DEGs among comparison groups (Figure 1). The number of DEGs co-expressed among comparison groups varied with each comparison (Figure 1), and 56 DEGs were shared among the five DEG datasets.

### 3.4. GO Enrichment Analysis of DEGs

**Figure 2:**
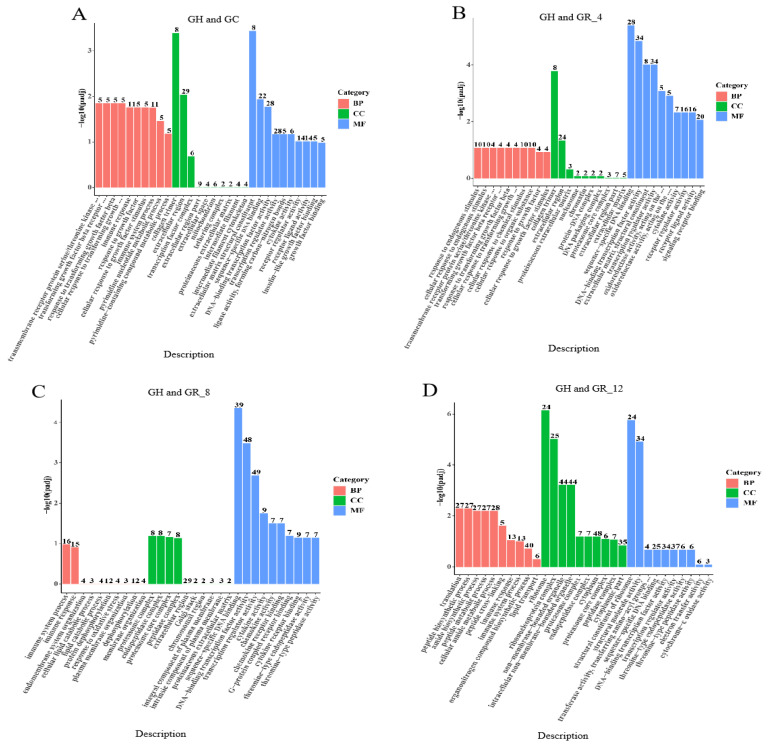

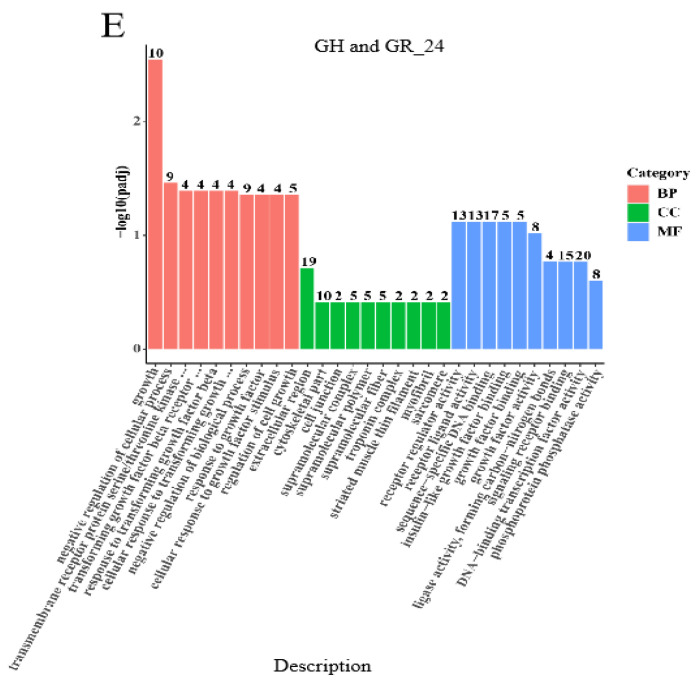
Gene Ontology (GO) annotation of significantly differentially expressed genes (padj < 0.05). GO annotations are shown for comparison groups (**A**) GH and GC, (**B**) GH and GR_4, (**C**) GH and GR_8, (**D**) GH and GR_12, and (**E**) GH and GR_24. The *X*-axis shows the GO term, while the *Y*-axis shows the statistical significance of GO term enrichment, expressed as −log10(padj). Different colors indicate different functional classifications.

The DEGs were functionally annotated via comparison to the Gene Ontology (GO) database. DEGs from each treatment group were also subjected to GO enrichment analysis and classified into three GO categories, namely, biological process (BP), cellular component (CC), and molecular function (MF). In the comparison of GC and GH (Figure 2), significant GO enrichment of DEGs (padj < 0.05) was observed at all three levels, but with higher enrichment for immune responses, immune system processes, and DNA binding transcription factor activity. In the GR_4 and GH comparison, significant GO enrichment of DEGs was observed for CC and MF groups, with significantly enriched categories corresponding to extracellular regions, DNA-binding transcription factor activity, and transcriptional regulator activity. In the GR_8 and GH comparison, a higher number of enriched DEGs was observed specifically for transcriptional regulator activity and DNA-binding transcription factor activity categories. For the GR_12 and GH comparison, the highest number of DEGs were enriched in non-membrane-bounded organelle and intracellular non-membrane-bounded organelle categories. Lastly, for the GR_24 and GH comparison, significant GO enrichment was only observed for the BP category, with significant enrichment of categories related to growth, negative regulation of cellular processes, and negative regulation of biological processes.

### 3.5. KEGG Enrichment Analysis of DEGs

**Figure 3:**
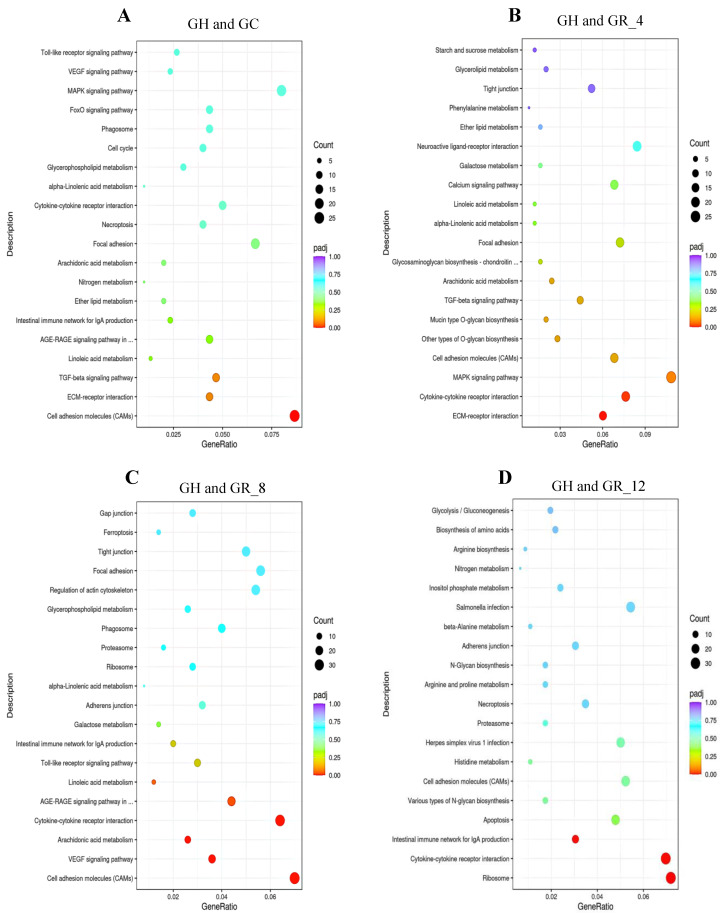

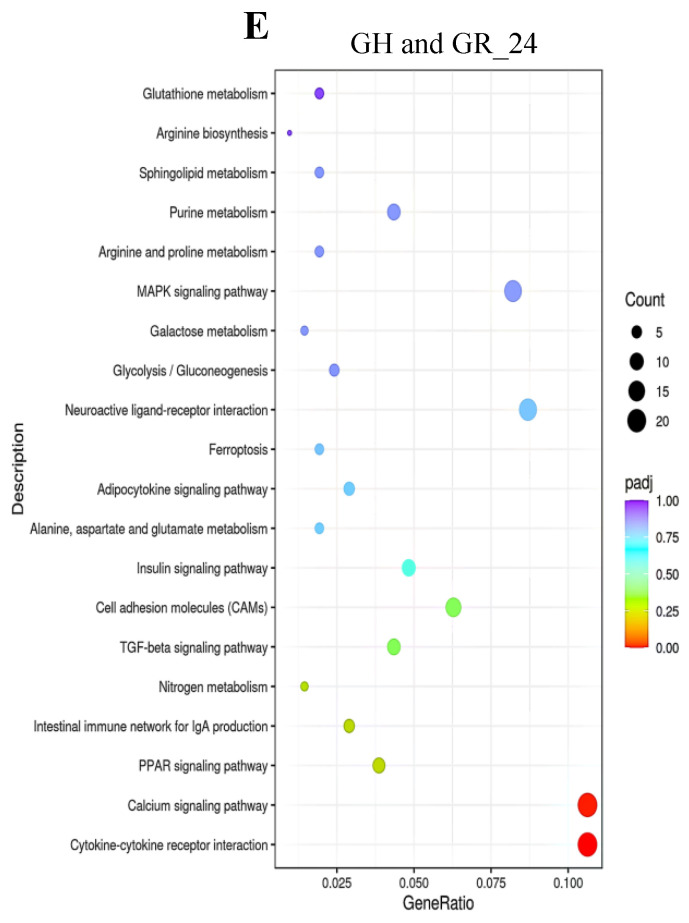
KEGG pathways most significantly enriched among differentially expressed genes (DEGs) between treatment groups. Pathways are shown for comparison groups GH and (**A**) GC, (**B**) GR_4, (**C**) GR_8, (**D**) GR_12, and (**E**) GR_24. Each circle is a KEGG pathway, and the *X*-axis shows the ratio of number of DEGs annotated to the total number of DEGs for the KEGG pathway, while the *Y*-axis shows the corresponding KEGG pathways. The color of the circle indicates the value of padj, and the size of the circle indicates the number of differential genes enriched by the pathway.

The KEGG database enables a holistic study of genes and their expression information through integrating data from multiple sources. Therefore, to further investigate the functions of DEGs expressed under acute hypoxic stress, KEGG enrichment analysis was performed (Figure 3). The KEGG pathway that differed most significantly between GH and GC transcriptomes was cell adhesion molecules (Figure 3A). In addition, the mitogen-activated protein kinase (MAPK) signaling pathway was the most enriched among DEGs between GR_4 and GH (Figure 3B). For the GR_8 and GH comparison, DEGs were significantly enriched in cell adhesion molecules (CAMs), cytokine–cytokine receptor interaction pathways, and the vascular endothelial growth factor (VEGF) signaling pathway (Figure 3C). Three significantly enriched pathways were identified in the GR_12 and GH comparison, including the ribosome, cytokine–cytokine receptor interaction, and intestinal immune network pathways for IgA production (Figure 3D). Lastly, the cytokine–cytokine receptor interaction and calcium signaling pathways were significantly enriched in the GR_24 and GH comparison (Figure 3E).

Overall, four pathways/functional categories that were closely related to hypoxia tolerance and were enriched in many of the DEGs during hypoxia were identified, including CAMs, cytokine–cytokine receptor interactions, the VEGF signaling pathway, and the MAPK signaling pathway. Thirty-four total DEGs were identified, comprising genes encoding bone morphogenetic protein 10 (*bmp10*), interleukin-8 (*il-8*), c-x-c motif chemokine receptor 4 (*cxcr4*), atypical chemokine receptor 3 (*ackr3*), vascular endothelial growth factor a (*vegfa*), prostaglandin-endoperoxide synthase 2 (*ptgs2*), and dual specificity phosphatase 1 (*dusp1*), which may be involved in regulating adaptations of *T. obscurus* to hypoxia (Table 4).

## 4. Discussion

Exposure to hypoxic environments can lead to respiratory and metabolic disturbances in fish, resulting in increased mortality [9]. Gills are primarily involved in respiration, osmoregulation, detoxification, immune functioning, and neuronal signaling, as well as being the first tissues in fish to be affected by a lack of oxygen in external environments [8]. To better understand the physiological mechanisms of acute hypoxic stress in *T. obscurus*, transcriptomic profiling of *T. obscurus* gill tissues was evaluated under acute hypoxic stress in this study, followed by the identification of DEGs and the functional annotation of pathways that were enriched in comparison groups and that may be involved in the regulation of hypoxic stress.

The number of DEGs was highest in the GR_8 vs. GH comparison group and lowest in the GR_24 vs. GH comparison group. The number of DEGs exhibited an increasing trend, followed by a decreasing trend with the extent of reoxygenation time, when compared to the hypoxia group. After 4, 8, and 12 h of reoxygenation recovery, the number of downregulated DEGs (844) was significantly higher than the number of upregulated DEGs (568), indicating that gill tissues alleviated the stress response via downregulating the expression of more genes during reoxygenation. There histograms were made for some of the important differential genes in the experiment at different time periods (Figure S1).

Specific analysis of the 56 DEGs present in all five DEG datasets revealed that most were expressed at low levels in the normoxic group but were significantly upregulated in the hypoxic group, followed by returns to expression levels similar to those under normoxic conditions after reoxygenation. Most of the genes were found to be focused on immune response and inflammatory response, while a small number of genes were involved in regulating cell proliferation and apoptosis, and others played roles in transcriptional regulation, cell development, and tissue differentiation. In particular, the expression levels of the tsc22 domain family member 3 (*tsc22d3*), cellular communication network factor 1 (*ccn1*), cellular communication network factor 2 (*ccn2*), bone morphogenetic protein 10 (*bmp10)*, and dual specificity phosphatase 1 (*dusp1)* genes were significantly higher under hypoxic conditions. *Tsc22d3* is a member of the TSC22 structural domain family and is referred to as a glucocorticoid-responsive molecule that interacts with intracellular signaling proteins that interact with and inhibit the activity of key inflammatory signaling mediators such as NF-κB and the activator protein-1 (AP-1). These processes ultimately regulate the transcription of inflammation-related genes and play a key role in anti-inflammatory and immunosuppressive activities [10]. Hypoxia can significantly upregulate the expression of *tsc22d3*, consistent with previous analyses [11] in rat spleens, where it significantly inhibited the expression of pro-inflammatory cytokines of macrophages. Under hypoxic stress, *tsc22d3* is hypothesized to exert a protective effect through inhibiting the overproduction of pro-inflammatory cytokines and preventing the overactivation of immune cells.

Both *ccn1* and *ccn2* play important roles in angiogenesis development, and both can induce angiogenesis by directly binding integrin αvβ3 on endothelial cells [12] or by regulating the expression of other angiogenic factors, such as VEGF [13]. The hypoxia treatments in this study were associated with significantly increased expression of *ccn1* and *ccn2*, suggesting that the organisms promoted angiogenesis via up regulating their expression. *Bmp10* is a bone morphogenetic protein with important roles in embryonic development, histogenesis, and reproductive regulation [14], in addition to the development, homeostatic balance, and repair of most organismal tissues [15]. Specifically, *bmp10* binds extracellularly as a ligand and transmembrane receptor, that in turn regulates the hydroxyl terminus of the Smads protein by activating it via phosphorylation and regulating the transcription of downstream genes. Other studies have shown that *bmp10* is highly expressed in fish gill tissues [16], consistent with the results of the up regulation of *bmp10* expression in the gills of fish in this study during hypoxia, which presumably contributes to the balance of dynamic physiological changes by helping *T. obscurus* resist hypoxic stress. *Dusp1* is a bispecific phosphatase 1 that mainly catalyzes the hydrolysis of specific phosphate groups in activated intracellular members of the MAPK family to achieve the inhibition of their activity, thereby blocking associated downstream biological responses [17]. Hypoxia has been shown to induce the upregulation of *dusp1* expression, consistent with previous results [18], wherein elevated DUSP expression was observed in mouse brain tissues after hypoxia. Moreover, these results are consistent with others [19], wherein the upregulation of *dusp1* mRNA was observed in mouse brain, liver, kidney, heart, and lung tissues. Lastly, *dusp1* was upregulated during hypoxia and encodes an enzyme that dephosphorylates and inactivates p38 [20], suggesting that it mitigates the onset of apoptosis in *T. obscurus* by inhibiting p38-related processes and protects the organism from cellular inflammatory immune responses.

GO enrichment analysis was performed with the DEGs, revealing that they were significantly enriched for categories including transmembrane receptor protein serine/threonine kinase signaling pathways, the signaling pathway of TGF-β, immune responses, negative regulation of cellular processes, collagen trimerization, extracellular regions, DNA-binding transcription factor activity, sequence-specific DNA binding, and transcriptional regulator activity. Among these significantly enriched categories, genes related to hypoxic regulation were identified, including those encoding Smad family member 6 (*smad6*), Smad family member 7 (*smad7*), *Bmp*, activin-membrane-bound inhibitor (*bambi*), interleukin 8 *(il-8)*, c-c motif chemokine ligand 20 (*ccl20*), and DNA-damage-inducible transcript 4 (*ddit4*). Of these, TGF-β plays a key role in cellular and tissue growth, development, and differentiation [21], while *smad6*, *smad7*, and *bambi* are all involved in the regulation of the TGF-β signaling pathway. Smads proteins transmit TGF-β signals from cell membranes directly to nuclei and are important intracellular TGF-β signal transduction and regulation molecules. *Smad6* and *smad7* encode inhibitory proteins within the Smads protein family, and both are negative regulators of this signaling pathway along with *bambi* [22], which inhibits the phosphorylation of R-Smads by binding to activated TβR-I type receptors, thereby blocking the signaling pathway and ultimately affecting the biological functions of TGF-β [23]. *Smad6*, *smad7*, and *bambi* were significantly upregulated at the onset of hypoxic stress in the experiments conducted in this study, inhibiting pathways from exerting their functions and hindering cell growth and development, while promoting apoptosis. In addition, TGF-β is an important promoter of immune homeostasis and immune tolerance. Ping [24] cloned TGF-β1 from head kidney lymphocytes of the oblique banded grouper and preliminarily showed that the function of TGF-β1 is related to immune mechanisms, with its immune function primarily related to immunosuppression. Specifically, TGF-β1 inhibits the proliferation and differentiation of T and B lymphocytes.

*IL-8* is an interleukin and is also referred to as *cxcl8*. It is primarily produced by monocyte-macrophages and is an important immune chemokine whose main function is to regulate immune activities. *IL-8* undergoes morphological changes upon contact with neutrophils and releases a series of active products that lead to a local inflammatory response, thereby achieving bactericidal and cellular damage effects [25]. Hypoxia has been shown to reduce *cxcl8* expression in cells [26], and *cxcl8* expression was downregulated during hypoxia in the present study. These results are consistent with those of previous studies [27] that evaluated the transcriptional responses of catfish to hypoxic environments, revealing the general downregulation of immunoreactive DEGs in hypoxic environments. The downregulation of *cxcl8* expression is mediated by HIF-1 and HIF-2, wherein HIF-1 decreases the expression of *Nrf2* via promoting the expression of *Bach1,* which ultimately regulates the expression of *cxcl8* [28]. In addition, *ccl20* is a chemokine whose sole ligand is c-c motif chemokine receptor 6 (*ccr6*), and it also plays an important role in autoimmune diseases. *Ccl20* exhibits almost no hydrophobic regions on its surface and has strong antimicrobial activity, while transgenic tumor cells of *ccl20* also inhibit tumor growth [29]. *Ccl20* acts as an important mediator in the initiation and effector phases of inflammatory responses through linking innate and acquired immunity [30]. *Ccl20* expression was also downregulated during the hypoxic phase of this study. These results suggest that hypoxia suppresses the immune function of fish in multiple ways, thereby increasing the risk of pathogenic bacterial infection of *T. obscurus*. The expression of *ddit4* is involved in key regulatory processes of epidermal cells under hypoxic conditions [31] and was significantly upregulated during hypoxic stress in this study. Consequently, the upregulation of *ddit4* may inhibit the migration ability of cells, thereby promoting apoptosis. In addition, the gill tissues of *T. obscurus* were significantly enriched in receptor-ligand-binding active functions under hypoxic stress, consistent with the outcomes of hypoxia tolerance in *Trachinotus blochii* [32]. Such activities may have beneficial effects on the immune functions of organisms in response to hypoxic stress in order to maintain normal physiological activities. Overall, it was apparent that hypoxic stress altered some processes related to growth and development, immune function, apoptosis, and the inflammatory responses of *T. obscurus*.

KEGG enrichment analysis further revealed that functional pathways significantly enriched among DEGs were primarily related to cell adhesion molecules (CAMs), cytokine–cytokine receptor interactions, the VEGF signaling pathway, and the MAPK signaling pathway. The DEGs related to these pathways were involved in immune functions, stress responses, angiogenesis, and apoptosis, thereby increasing host adaptability to hypoxia and the survival of *T. obscurus*. CAMs that exhibit receptor–ligand binding activities are involved in cellular recognition, activation, and signal transduction. Cytokines are involved in cell growth and differentiation, cell death, and adaptive inflammatory host defenses, and related studies show that hypoxic stimulation induces cytokines that regulate homeostatic development and regulate the equilibrium processes in vivo [33]. The cytokine–cytokine receptor interaction pathway plays an important regulatory role in the immune defense of fish. In the present study, most of the DEGs in this pathway with significant changes in expression during the phases of hypoxia reoxygenation were immune factors, including the chemokines *cxcr4*, c-x-c motif chemokine receptor 5 (*cxcr5*), c-x-c motif chemokine ligand 14 (*cxcl14*), and atypical chemokine receptor 3 (*ackr3*), as well as the interleukin 8 (*il-8)*, interleukin 11 (*il-11*), and interleukin 17 (*il-17*). The differential expression of these cytokines during hypoxia is essential for mediating various inflammatory and immune responses. In addition, the VEGF signaling pathway also plays an important role in hypoxia, especially because it has been shown that acute hypoxia can lead to increased VEGF expression [34], which can promote angiogenesis, increase oxygen transport in vivo through regulating cell membrane permeability, and improve the hypoxic tolerances of organisms. *Vegfa* is the most important factor of the VEGF family, and its expression was increased during the experimental hypoxic stress phase compared to normoxic conditions, followed by gradual decreases in expression with reoxygenation. These results are consistent with hypoxic conditions increasing VEGF expression in *Oncorhynchus* [35]. Moreover, these results are consistent with the findings of previous research [36] that hypoxia increases VEGF expression and stimulates angiogenesis, such that VEGF expression in endothelial cells decreases after improved tissue oxygenation. Thus, the increased *vegfa* expression in *T. obscurus* may be due to the upregulation of VEGF expression to stimulate angiogenesis and increase vascular permeability, thereby increasing physiological oxygen and nutrient levels to promote adaptation to hypoxic environments. Similarly, the gradual decrease in *vegfa* expression after reoxygenation may be due to the restoration of oxygen supply. Consequently, the expression of *vegfa* may be an important response to acute hypoxic stress in *T. obscurus* and may be able to be used as an indicator of their response to hypoxic stress. Other studies have shown that *vegf* also regulates inflammatory responses, in addition to regulating oxygen supply and angiogenesis. In hypoxic environments, *vegf* can bind to VEGF receptors on endothelial cell membranes, causing receptor autophosphorylation [37]. In this study, hypoxia stress may have significantly increased the expression of *vegf* and *ptgs2*, thus causing oxidative stress, immunosuppression, and endocrine disorders in *T. obscurus*. The MAPK signaling pathway was enriched with higher numbers of DEGs in each DEG comparison group. The MAPK family includes enzymes involved in oxidative sensing and plays important roles in coordinating the metabolic and energetic homeostasis of organisms through participating in inflammatory and apoptotic stress responses. The MAPK pathway primarily comprises extracellular signal-regulated kinases (ERKs), c-Jun amino-terminal kinases (JNKs), and p38 kinase pathways, with their activity regulated by the ratio of ATP to AMP [34]. In this study, hypoxia activated the MAPK pathway through inhibiting ATP production, thereby affecting the ratio of ATP to AMP. Consequently, the MAPK signaling pathway may be involved in the regulation of hypoxic adaptations in *T. obscurus*. Hypoxic stress experiments in *Danio rerio* have also revealed that the expression of MAPKs can be altered and consequently regulate the associated signaling pathways [38].

## 5. Conclusions

To understand the physiological adaptations of *T. obscurus* during acute DO availability changes, the transcriptomic profiles of *T. obscurus* gill tissues were evaluated under normal oxygen, hypoxia stress, and reoxygenation conditions. DEGs under hypoxia stress were associated with CAMs, cytokine–cytokine receptor interactions, the VEGF signaling pathway, and the MAPK signaling pathway. Most of the DEGs in gills under hypoxic stress mainly play a role in immune response, and a small proportion of these DEGs are involved in cell growth and development, as well as in angiogenesis and apoptosis processes. It was also found that changes in the expression of genes including *il-8*, *il-11*, *il-17*, *cxcr4*, *cxcr5*, *cxcl14*, and *ccl20* were suppressed under hypoxic stress, leading to decreased immunity in fish; growth and development related genes including *smad6*, *smad7,* and *bambi* hindered cell growth and development; and genes including *dusp1* and *ddit4* regulated the apoptotic process in gills. These results can help to identify genes associated with hypoxia stress and elucidate the mechanism through which *T. obscurus* adapts to hypoxia, thereby providing a framework for further research into the molecular mechanisms of hypoxia tolerance in other fish.

## Figures and Tables

**Table 1:** Summary of sample sequencing data quality.

Sample	Raw Reads	Raw Bases	Clean Reads	Clean Bases	Error Rate	GC Content (%)	Q30 (%)
GC1	43,559,868	6.53 G	41,654,646	6.25 G	0.03	50.49	92.97
GC2	46,829,616	7.02 G	42,629,596	6.39 G	0.03	49.92	93.64
GC3	46,140,914	6.92 G	42,115,486	6.32 G	0.03	50.10	93.85
GH1	45,868,852	6.88 G	42,349,752	6.35 G	0.03	49.94	93.61
GH2	46,227,668	6.93 G	44,462,882	6.67 G	0.03	50.62	93.41
GH3	45,886,854	6.88 G	43,746,966	6.56 G	0.03	50.64	93.14
GR1_4	45,403,326	6.81 G	42,937,580	6.44 G	0.03	50.69	93.36
GR2_4	42,998,268	6.45 G	40,784,166	6.12 G	0.03	50.88	93.25
GR3_4	45,506,236	6.83 G	43,479,216	6.52 G	0.03	51.25	93.10
GR1_8	45,482,486	6.82 G	42,964,718	6.44 G	0.03	50.91	93.29
GR2_8	46,235,922	6.94 G	43,898,446	6.58 G	0.03	51.01	93.15
GR3_8	47,141,580	7.07 G	43,991,278	6.60 G	0.03	50.65	93.47
GR1_12	44,758,330	6.71 G	40,769,722	6.12 G	0.03	50.28	93.32
GR2_12	47,797,410	7.17 G	44,347,812	6.65 G	0.03	49.87	93.05
GR3_12	42,904,626	6.44 G	39,202,820	5.88 G	0.03	50.52	93.29
GR1_24	46,838,548	7.03 G	45,699,722	6.85 G	0.03	49.39	93.30
GR2_24	46,183,646	6.93 G	44,378,028	6.66 G	0.03	50.60	93.07
GR3_24	46,388,604	6.96 G	44,297,968	6.64 G	0.03	51.02	93.15

Notes: sample: name of sample; raw reads: number of reads in raw data; raw bases: number of bases in raw data; clean reads: the number of reads remaining after quality filtering; clean bases: the number of bases in the dataset after quality filtering the dataset; error rate: overall sequencing error rate for the dataset; GC content: the percentage of G and C base pairs among the datasets; Q30: the percentage of bases with Phred values > 30 among the total datasets.

**Table 2:** Statistics on the comparison of samples with the reference genome.

Sample	Total Map	Unique Map	Multi Map
GC1	36,656,072 (88.0%)	33,274,418 (79.88%)	3,381,654 (8.12%)
GC2	36,636,834 (85.94%)	33,391,350 (78.33%)	3,245,484 (7.61%)
GC3	36,675,924 (87.08%)	33,628,770 (79.85%)	3,047,154 (7.24%)
GH1	36,779,197 (86.85%)	34,025,598 (80.34%)	2,753,599 (6.5%)
GH2	38,854,740 (87.39%)	36,075,442 (81.14%)	2,779,298 (6.25%)
GH3	38,481,642 (87.96%)	35,928,246 (82.13%)	2,553,396 (5.84%)
GR1_4	37,403,666 (87.11%)	34,038,280 (79.27%)	3,365,386 (7.84%)
GR2_4	35,918,644 (88.07%)	32,457,035 (79.58%)	3,461,609 (8.49%)
GR3_4	37,420,039 (86.06%)	33,074,820 (76.07%)	4,345,219 (9.99%)
GR1_8	37,468,050 (87.21%)	34,102,188 (79.37%)	3,365,862 (7.83%)
GR2_8	38,591,719 (87.91%)	35,161,827 (80.1%)	3,429,892 (7.81%)
GR3_8	38,103,192 (86.62%)	35,332,509 (80.32%)	2,770,683 (6.3%)
GR1_12	35,190,781 (86.32%)	32,123,707 (78.79%)	3,067,074 (7.52%)
GR2_12	38,460,120 (86.72%)	34,765,756 (78.39%)	3,694,364 (8.33%)
GR3_12	32,694,236 (83.4%)	29,370,781 (74.92%)	3,323,455 (8.48%)
GR1_24	39,216,187 (85.81%)	36,171,393 (79.15%)	3,044,794 (6.66%)
GR2_24	38,553,073 (86.87%)	35,856,894 (80.8%)	2,696,179 (6.08%)
GR3_24	37,729,741 (85.17%)	34,958,208 (78.92%)	2,771,533 (6.26%)

Notes: sample: name of sample; total map: number of reads compared to the genome and percentage; unique map: number of reads compared to the unique position of the reference genome and percentage; multi map: number of reads compared to multiple positions in the reference genome and percentage.

**Table 3:** Differentially expressed gene (DEG) summaries.

DEG Comparison	DEG Number	Upregulated Genes	Downregulated Genes
GC vs. GH	992	426	566
GR_4 vs. GH	877	405	472
GR_8 vs. GH	1561	758	803
GR_12 vs. GH	1412	568	844
GR_24 vs. GH	679	325	354

Notes: DEG comparison: comparisons of DEGs among treatment groups; DEG number: the number of DEGs; upregulated genes: the number of upregulated genes; downregulated genes: the number of downregulated genes.

**Table 4:** KEGG signaling pathways that were significantly enriched among differentially expressed genes in gill tissues.

KEGG ID Pathway	Signaling Pathway	*p*	Genes
tru04514	Cell adhesion molecules (CAMs)	0.000013	*nrxn1*, *cldn33b*, *ptprm*, *itga8, vtcn1*, *itgb8*, *LOC115249662*, *LOC101064604*, *LOC101063645*, *LOC101063580*
tru04060	Cytokine–cytokine receptor interaction	0.009122	*bmp10*, *il-8*, *cxcr4*, *ackr3*, *amh*, *il2rg*, *LOC115248669, LOC101066952*, *LOC101063250*
tru04370	VEGF signaling pathway	0.04077	*vegfa*, *ptgs2*, *pla2g4a*, *pik3r3*, *LOC101073653, LOC101070662*, *LOC101069259*
tru04010	MAPK signaling pathway	0.04903	*dusp1, igf1*, *ntrk2*, *LOC101077057*, *LOC101065285*, *LOC101076406*, *LOC10106965*, *LOC101067669*

## Data Availability

The data presented in this study are available in this article.

## Supplementary Materials

The following supporting information can be downloaded at: https://www.mdpi.com/article/10.3390/ani13101572/s1, Figure S1. Graph of partial differential change genes at different time periods.
