# Supplementary material for: Transcriptomic Analysis of Takifugu obscurus Gills under Acute Hypoxic Stress
Source: Animals (Basel). 2023 May 9;13(10):1572. doi: 10.3390/ani13101572 (PMC10215208; doi:10.3390/ani13101572)
Supplement: Supplementary file 1 [file animals-13-01572-s001.zip › animals-2298362-supplementary.pdf]

Supplementary Material

Transcriptomic Analysis of *Takifugu obscurus* Gills under Acute Hypoxic Stress

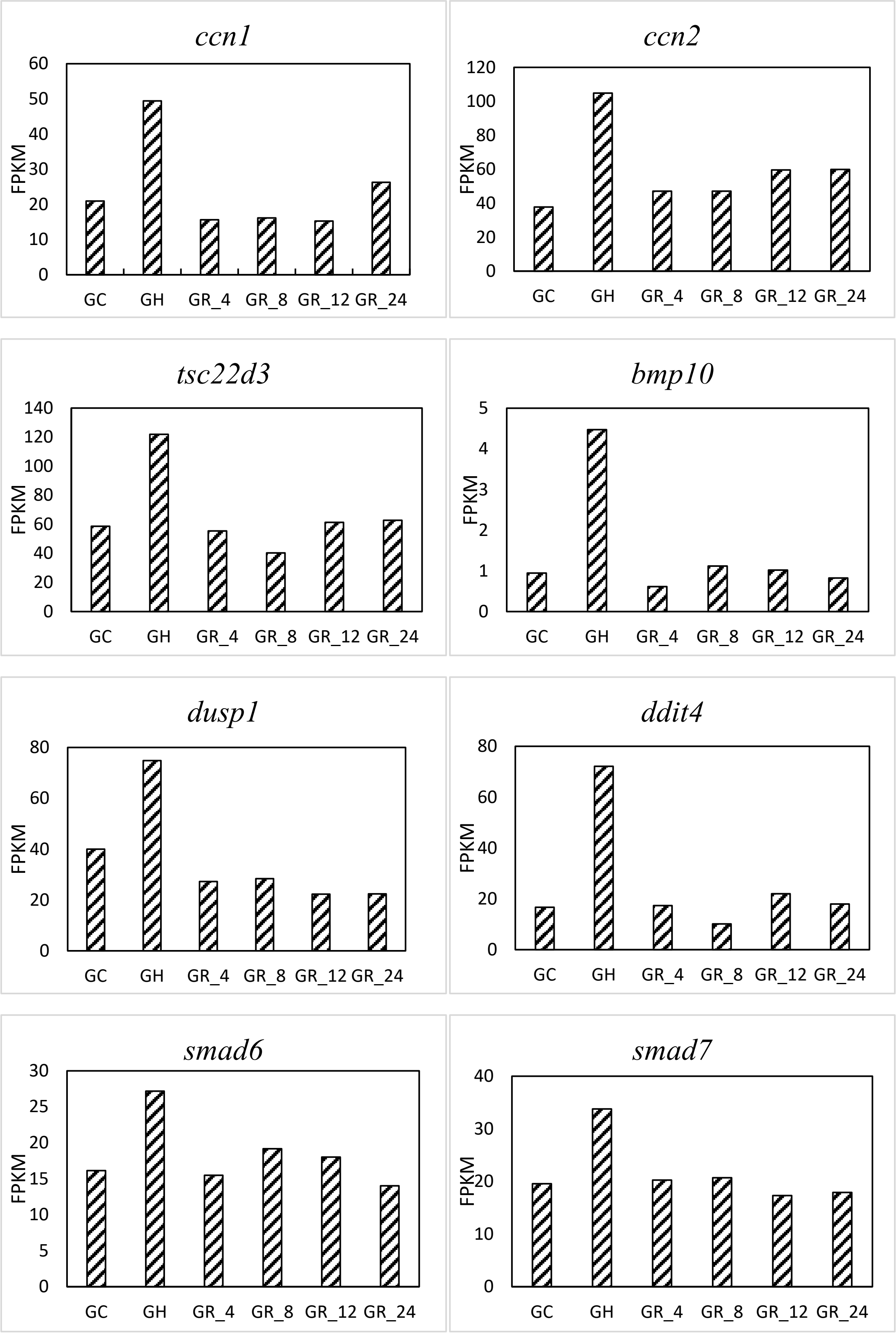

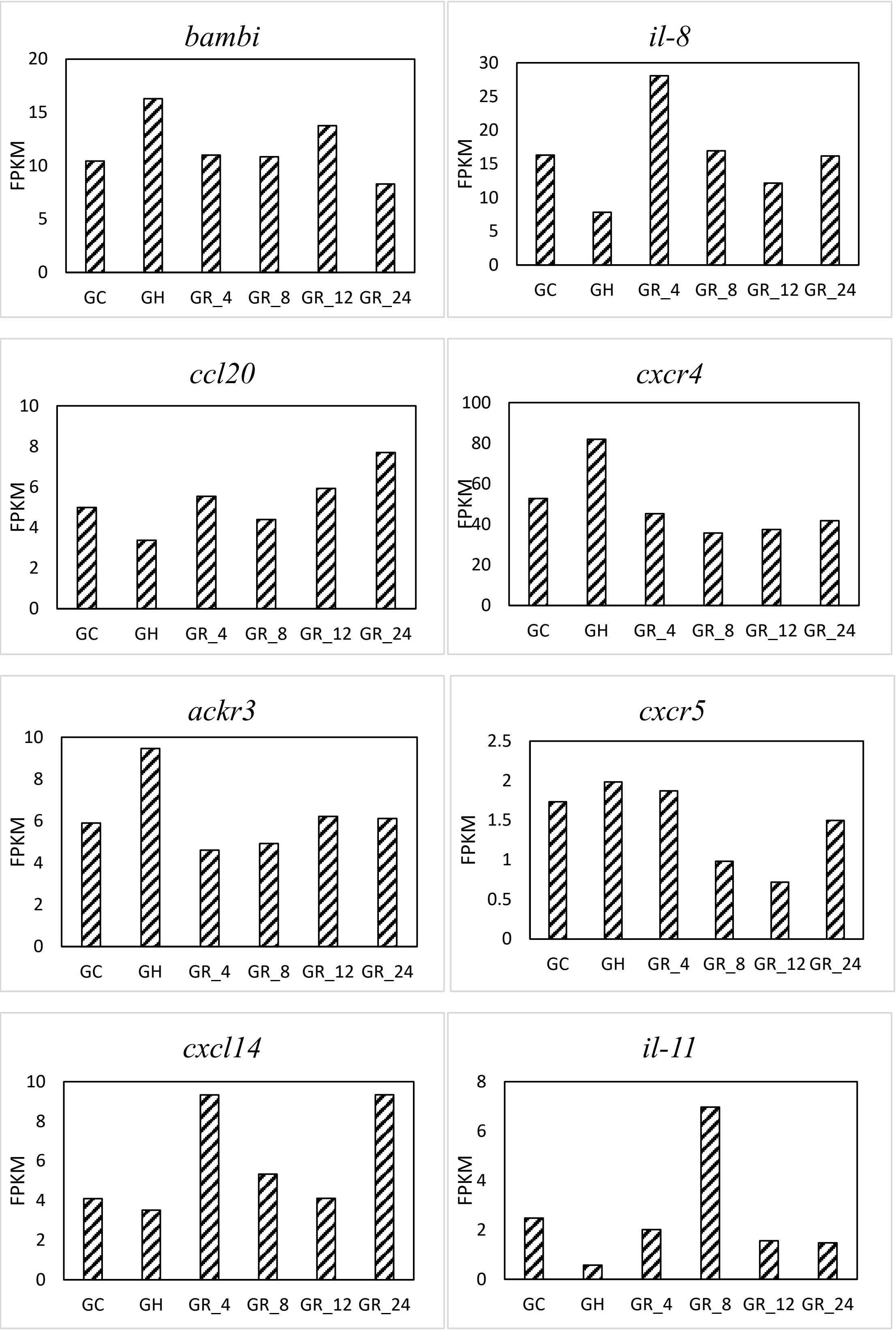
bambi
FPKM
0
5
10
15
20
GC
GH
GR_4
GR_8
GR_12
GR_24
il-8
FPKM
0
5
10
15
20
25
30
GC
GH
GR_4
GR_8
GR_12
GR_24
ccl20
FPKM
0
2
4
6
8
10
GC
GH
GR_4
GR_8
GR_12
GR_24
cxcr4
FPKM
0
20
40
60
80
100
GC
GH
GR_4
GR_8
GR_12
GR_24
ackr3
FPKM
0
2
4
6
8
10
GC
GH
GR_4
GR_8
GR_12
GR_24
cxcr5
FPKM
0
0.5
1
1.5
2
2.5
GC
GH
GR_4
GR_8
GR_12
GR_24
cxcl14
FPKM
0
2
4
6
8
10
GC
GH
GR_4
GR_8
GR_12
GR_24
il-11
FPKM
0
2
4
6
8
GC
GH
GR_4
GR_8
GR_12
GR_24

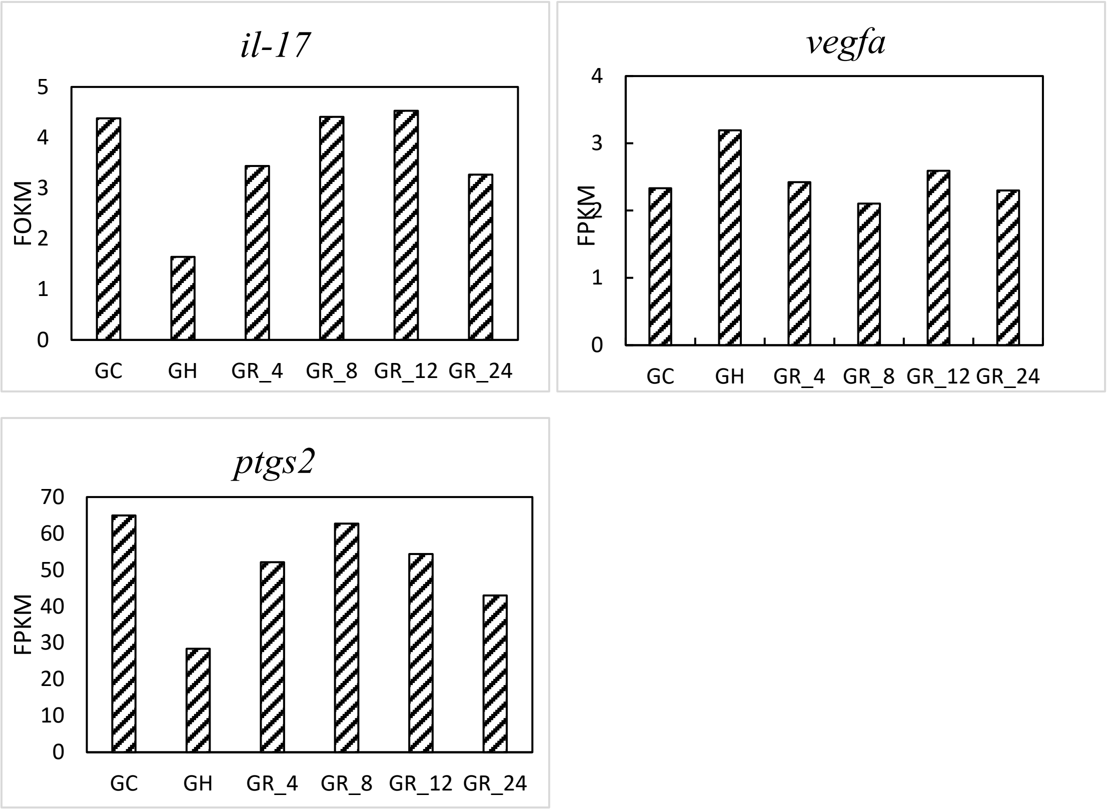

Figure S1. Graph of partial differential change genes at different time periods
